# Feasibility and validity of The Health Improvement Network database of primary care electronic health records to identify and characterise patients with small cell lung cancer in the United Kingdom

**DOI:** 10.1186/s12885-019-5305-1

**Published:** 2019-01-21

**Authors:** Lucía Cea Soriano, Jihong Zong, Luis A. García Rodríguez

**Affiliations:** 1 Spanish Centre for Pharmacoepidemiological Research (CEIFE), Almirante 28, 28004 Madrid, Spain; 2 Department of Public Health and Maternal and Child Health, Faculty of Medicine, Complutense University of Madrid, Madrid, Spain; 3 Epidemiology, Bayer Healthcare Pharmaceuticals Inc, Whippany, USA

**Keywords:** Small cell lung carcinoma, Database, Incidence

## Abstract

**Background:**

Epidemiological research on small cell lung cancer (SCLC) is limited and based on cancer registry data. We evaluated the feasibility and validity of using primary care electronic health records (The Health Improvement Network [THIN]) in the UK to identify and characterise SCLC.

**Methods:**

We searched THIN records of individuals aged 18–89 years between 2000 and 2014 for a first diagnostic code suggestive of lung cancer (group 1) or small cell cancer (SCC; group 2) and for text strings among free text comments to identify and characterise incident SCLC cases. We validated our case identification strategy by manual review of patient EHRs, including free text comments, for a random sample of 400 individuals initially detected with a diagnostic code (300 from group 1 and 100 from group 2).

**Results:**

Twenty five thousand two hundred fourty one individuals had a code for lung cancer (*n* = 24,508 [97.1%]) or SCC (733 [2.9%]). Following free-text searches, there were 3530 incident SCLC cases (2956 from group 1; 574 from group 2) corresponding to an incidence rate of 1.01 per 10,000 person-years. In the validation exercise, SCLC confirmation rates were 99% (group 1) and 85% (group 2). Mean age at diagnosis among confirmed cases was 68.5 years; staging information was present in 63.5% of cases of whom 17.8% had limited disease and 82.2% had extensive disease. The majority (84.5%) had a recorded symptom suggestive of lung cancer; chest infection was the most common (18%) followed by cough (15.8%) and chest/abdominal/back pain (15.2%). The first year crude mortality rates was 9.9 per 100 person-months (95% confidence interval [CI] 9.5–10.4), was higher among men and those aged 80 years and above. A total of 144 (37.8%) confirmed cases had metastases recorded. Median survival among the whole study cohort was 7.37 months.

**Conclusions:**

Our SCLC case identification method appears to be valid and could potentially be adapted to identify other cancer types. However, complete characterisation of staging requires information from additional data sources including cancer registries.

**Electronic supplementary material:**

The online version of this article (10.1186/s12885-019-5305-1) contains supplementary material, which is available to authorized users.

## Background

In Europe, lung cancer is the second most commonly diagnosed cancer in men and the third most commonly diagnosed cancer in women, with age-adjusted incidence rates in 2018 of 98.6 and 38.3 per 100,000 persons, respectively. [1] Approximately 10–15% of lung cancer cases are small cell lung cancer (SCLC), [2–5] an aggressive subtype associated with poor survival [6, 7]. Small cell lung cancer is both histologically and clinically distinct from non-small cell lung cancer (NSCLC), and patients are classed as having either extensive or limited disease. [2, 3] Symptoms often present rapidly in patients with SCLC and the majority have extensive disease at the time of diagnosis. In the UK, recommended first-line treatment is chemotherapy/chemoradiotherapy for patients with limited disease and chemotherapy alone for patients with extensive disease, dependent on performance status and comorbidity for both stages. [8]

Previous epidemiological research on SCLC is limited and mainly based on population registry data, [2, 4, 6, 9, 10] which may lack information on patient characteristics such as comorbidities and lifestyle factors. We aimed to evaluate the feasibility and validity of a population-based primary care database of electronic health records (EHRs) in the UK – The Health Improvement Network – to identify and characterise patients with SCLC including stage, comorbidities, treatment received, comedications and survival. To identify SCLC cases, we used computer algorithms involving text data mining using text strings.

## Methods

### Data source

The Health Improvement Network is a database containing the anonymized primary care EHRs of almost 3.1 million patients collected from over 385 general practices across the UK. Patient data are entered by primary care practitioners (PCPs) as part of routine care using Vision patient management software (In Practice Systems Ltd). Information recorded includes diagnoses, symptoms, referrals to secondary care and results of laboratory tests. Clinical data entries are coded using the Read classification system, [11] which have been used by the UK’s National Health Service (NHS) since 1985. Prescriptions are entered using GEMSCRIPT codes from the NHS drug dictionary [12] and are automatically recorded upon issue. For each clinical entry, a free text field enables PCPs to add additional details of relevance. Information received from secondary care regarding patient visits and admissions is also added to patients’ primary care records.

### Study cohort

We identified all individuals aged 18–89 years in THIN between January 2000 and December 2014 with a registration status of permanent or died and no previous diagnosis of lung cancer. To be included in the study cohort, individuals were required to have at least 2 years’ enrolment with the PCP, at least 2 years since their first recorded prescription and at least one encounter/visit recorded in the last 2 years. Cohort members were followed from the date these eligibility criteria were met (start date) until the earliest of the following: a first Read code for lung cancer or small cell cancer of unspecified type (**see Section 2.3**), death or the end of the study period (31 December 2014). It should be noted that no specific Read codes for SCLC exist, but there are Read codes for lung cancer (non-specific or specifically for NSCLC) and for small-cell cancer. The study protocol was approved by the independent scientific review committee for The Health Improvement Network (THIN) (reference number 15THIN077).

### Identification of SCLC cases

**Fig. 1:**
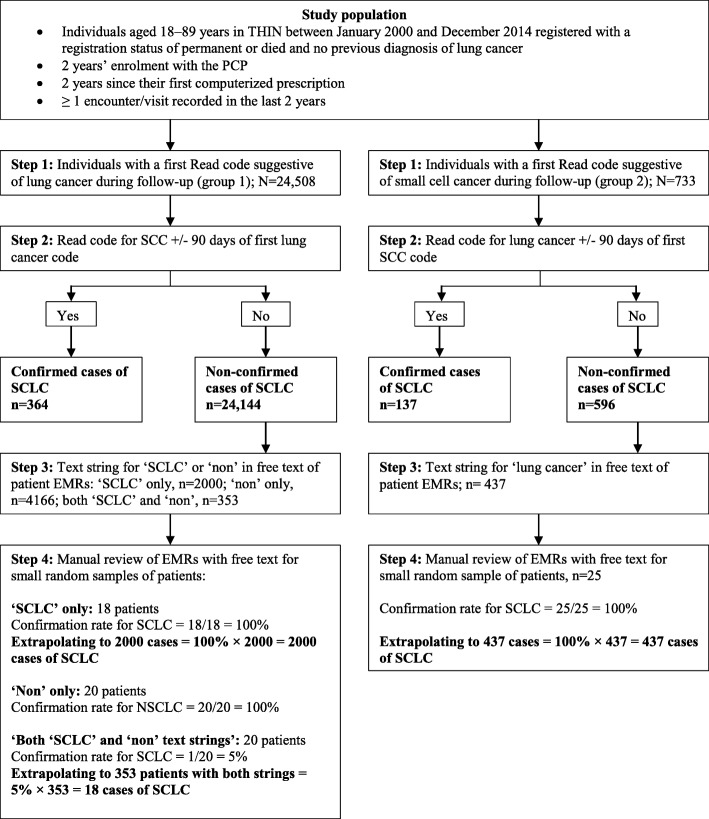
Flowchart depicting the SCLC case ascertainment

The strategy to identify incident cases of SCLC is depicted in Fig. 1. The first step involved automated computer searches of patients’ EHRs during follow-up for either a Read code suggestive of lung cancer (group 1; *N* = 24,508) or a Read code for small cell cancer (SCC; group 2; *N* = 733); see Additional file 1: Table S1 for the code list. Secondly, among individuals in group 1, we performed further computer searches of their medical records for entries of a SCC Read code within 90 days either side of the lung cancer code entry (*n* = 364, 1.5%), and vice versa (i.e. for group 2, we searched for a lung cancer code within 90 days of the SCC code entry; *n* = 137, 18.7%). We subsequently retained these 501 individuals as confirmed cases. Thirdly, for remaining patients not designated as confirmed cases from this process, (*n* = 24,144 [98.5%] in the lung cancer group and 596 [81.3%] in the SCC group), we requested the staff at THIN to search the free text comments among these individuals’ EHRs in their in-house version of the database for specific text strings suggestive of SCLC or lung cancer (as appropriate for both groups) using a time frame of 30 days before the initial computer-detected Read code entry to 90 days after. Although more sophisticated methods exist for text data mining, we chose to use this method of searching for specific text strings for simplicity. To avoid misclassification, searches for text strings suggestive of SCLC with ‘non’ preceding the entry were additionally performed for group 1 in order to exclude cases of NSCLC. In the lung cancer group, there were 2000 patients with a text string in the free text comments suggestive of SCLC, 4166 patients with text strings suggestive of ‘non’ and 353 with both ‘SCLC’ and ‘non’ text strings. Among group 2, there were 437 patients with a text string for ‘lung cancer’ in the free text comments. Fourthly, we selected small random samples of patients: 18/2000 group 1 individuals with only a ‘SCLC’ text string, 18/4166 group 1 individuals with only a ‘non’ text string, 20/353 group 1 individuals with both a ‘SCC’ and ‘non’ text strings, and 25/437 group 2 individuals with a ‘lung cancer’ text string. We manually reviewed these patients’ EHRs including the free text comments containing the detected text strings. Confirmation rates of SCLC were as follows: 100% for group 1 ‘SCLC’ text only, 5% for group 1 both ‘SCLC’ and ‘non’, and 100% for group 2 ‘lung cancer’ text. Also, there was 100% confirmation of NSCLC for group 1 ‘non’ only.

### Estimation of expected final number SCLC cases

As shown in Fig. 1, and to calculate the expected incidence of SCLC, we subsequently applied these confirmation rates to the total number of patients identified (in step 3) with these text strings to estimate the expected number of SCLC. There were a total of 2956 expected cases: from group 1, 364 + 2000 + 18 (from applying a 5% confirmation rate to those with both a ‘SCLC’ and ‘non’ text string’) and from group 2, 137 + 437. Overall, of the initial 25,241 patients (24,508 + 733) with a first computer-detected code for lung cancer or SCC during follow-up, 11.7% (2956/ 25,241) were classed as incident cases of SCLC. However, for further analyses we did not include the estimated 18 cases of SCLC that derived from the 353 patients detected with both a ‘SCLC’ and ‘non’ text string’ (in Step 3) in our final number of expected cases (*N* = 2938).

### Characterisation of all patients with newly diagnosed SCLC

For all 2938 cases of SCLC, we obtained information on their demographic and lifestyle factors (smoking, alcohol consumption and body mass index [BMI, kg/m^2^], comorbidities, medication and healthcare use using information in the database before the recorded SCLC diagnosis. For lifestyle factors, we used the status/value closest to the date of diagnosis; for medications, we considered current use to be when the supply of the most recent prescription lasted until the date of diagnosis or ended in the previous year.

### Case validation and disease characterisation

To further validate our SCLC case ascertainment strategy and to obtain information to characterise cases of SCLC, for a sample of 400 patients (300/2000 cases from group 1 and 100/437 cases from group 2), we undertook a manual review of their EHRs including all free text comments that were entered within 30 days either side of the recorded cancer date. We also reviewed all free text comments that were entered alongside Read coded entries of lung cancer, test procedures (respiratory system procedures such as chest X-ray and bronchoscopy, CAT and MRI scans), referrals, follow-up visits and terminal care suggestive of cancer, which were recorded between 30 days before the date of computer-detected cancer code and to 1 year after (see Additional file 1: Table S2 for Read codes). During this process, we considered a confirmed case to be a patient whose diagnosis resulted from biopsy. We excluded cases whose diagnosis was revealed to be before the start of follow-up (prevalent cases), was lung cancer other than SCLC, or was not in the lung but had small cell histology (e.g. misclassification of a renal cancer). We collected data on symptoms recorded near to the SCLC diagnosis (e.g. haemoptysis, chest infection, cough), stage of SCLC (extensive or limited based either on free text notes containing descriptive text such as ‘extensive’, ‘limited’, ‘spread to lymph nodes’ ‘occurrence of metastases’ or actual TNM stage), presence of metastasis at the time of diagnosis (and, where present, the affected organ), diagnostic technique used for SCLC detection (chest X-ray, bronchoscopy, CAT scan and adjuvant therapy (chemotherapy and/or radiotherapy). Of note is that while there are some specific codes suggestive of chemotherapy and radiotherapy in THIN, details are most commonly entered as free text comments. We assigned the final SCLC event date as either the date of the first related symptom, diagnostic procedure or surgery, whichever came earlier.

### One-year all-cause mortality

We followed all SCLC cases for 1 year to identify deaths. These were identified using automatic computer searches for relevant Read codes, recording of death certification or if the patient had a registration status of died. Owing to updates to the database or change in patients’ registration status, registration not all patients (*n* = 98) were included in the all-cause mortality analysis.

### Statistical analysis

Incidence rates of SCLC with 95% confidence intervals (CIs) were calculated as the number of incident SCLC cases divided by the total person-years. Baseline characteristics of SCLC cases were described. Categorical variables were presented as frequency counts and percentages. Age at diagnosis was described using the mean (± standard deviation [SD]) and median (interquartile range [IQR]). Among the sample of 400 SCLC cases for whom free text were requested (using our final confirmed case status following the case validation process as gold standard), we calculated the positive predictive value (PPV) of the recorded SCLC diagnoses in THIN (following our four-step case ascertainment strategy). One-year all-cause mortality rates and 1-year cumulative survival was calculated and Kaplan–Meier cumulative survival curves were produced. Statistical analyses were performed using Stata version 12.0 (StataCorp LP, College Station, TX, US).

## Results

### Expected incidence of SCLC and patient characteristics

Based on the total estimated number of 2956 incident cases of SCLC occurring within a total of 29,028,641 person-years (median 202 days, interquartile range: 64.5–365 days), we estimated an expected incidence rate of SCLC of 1.01 cases per 10,000 person-years. Baseline characteristics of SCLC cases are shown in Table 1. Ninety four percent of SCLC cases were current of former smokers and one third were overweight. The most frequent comorbidities were those of the respiratory tract or the digestive system. Use of respiratory medications in the year before SCLC diagnosis was high with beta-2 agonists present in 40.0% (a quarter of beta-2 agonists users had used the medication for more than 1 year). More than half of SCLC cases had at least one prescription for opioids within the year before their cancer diagnosis, with two thirds of these patients having a recorded use of less than 3 months.

**Table 1:** Baseline characteristics of incident SCLC cases

Baseline characteristic	Incident SCLC cases (*N* = 2938)	
Sex	n	%
Male	1524	51.9
Female	1414	48.1
Age (years)		
Mean (±SD)	68.45 (68.10–68.80)	
50–59	94	3.2
60–69	451	15.4
70–79	972	33.1
80–89	1054	35.9
Smoking^a^		
Non-smoker	141	4.8
Current	1455	49.5
Former	1299	44.2
Unknown	43	1.5
BMI (kg/m^2^)^a^		
15–19	189	6.4
20–24	945	32.2
25–29	926	31.5
≥30	590	20.1
Unknown	288	9.8
Alcohol (units/week)^a^		
None	18,790	16.5
1–9	47,121	41.4
10–20	14,390	12.6
21–41	4986	4.4
≥42	1820	1.6
Unknown	26,833	23.6
Geographical setting		
Urban	1923	65.5
Town	303	10.3
Rural	113	3.8
Unknown	599	20.4
Comorbidities^b^		
MI	285	9.7
IS	153	5.2
PAD	289	9.8
Heart failure	152	5.2
Hypertension	1256	42.8
Hyperlipidaemia	646	22.0
Dyspepsia	789	26.9
Depression	847	28.8
GERD	531	18.1
Asthma	718	24.4
COPD	739	25.2
Medications^c^		
Beta2-agonists	1181	40.2
Oral corticosteroids	849	28.9
Inhaled steroids	624	21.2
Antiplatelets	1097	37.3
NSAIDs	810	27.6
Opioids	1604	54.6
PPIs	1125	38.3
H_2_RA	232	7.9

^a^Ever before SCLC diagnosis using the most recent value/status as appropriate

^b^Ever before SCLC diagnosis

^c^In the year prior to SCLC diagnosis

*BMI* body mass index, *COPD* chronic obstructive pulmonary disease, *DVT* deep vein thrombosis, *GERD* gastro-oesophageal reflux disease, *H*_*2*_*RA* histamine 2 receptor antagonists, *IS* ischaemic stroke, *MI* myocardial infarction, *NSAID* non-steroidal anti-inflammatory drug, *PAD* peripheral artery disease, *PPI* proton pump inhibitor, *SCLC* small-cell lung cancer, *SD* standard deviation, *TIA* transient ischaemic attack

### Disease characterisation among validated cases

Among the sample of 400 patients for whom we manually reviewed their EHRs including free text comments (300 cases from group 1, and 100 cases from group 2), 381 were confirmed as incident SCLC cases (296/300 in group 1 and 85/100 in group 2; see Additional file 1: Table S3), corresponding to a PPV of 95.2% (98.7% in the lung cancer group and 85.0% in the SCC group). Positive predictive values according age, sex and smoking status can be found in Additional file 1: Table S4). Of the 19 patients not confirmed as incident cases of SCLC, the reasons were as follows: cases of NSCLC (*n* = 14), lung cancer was a secondary tumour (*n* = 2), prevalent case (*n* = 1), unconfirmed based on the data recorded (*n* = 1), tumour was in a different location (*n* = 1). The SCLC event date was backdated from the date of the recorded diagnosis in the majority of patients (83%); the median number of backdated days was 36. Mean age at SCLC diagnosis date was 69.0 year (SD: ± 9.9). Disease characteristics among the 381 incident SCLC cases are shown in Table 2. The majority (84.5%) had a recorded symptom suggestive of lung cancer; chest infection was the most common (18%) followed by cough (15.8%) and chest/abdominal/back pain (15.2%); almost two-thirds of cases (65.5%) had a record of chest X-ray as the first diagnostic procedure. Stage of disease was recorded in 63.5% of confirmed cases and, of these, 17.8% had limited stage and 82.2% extensive stage disease. Fifty six per cent of cases had a record of chemotherapy and 32% had a record of radiotherapy. A total of 144 (37.8%) confirmed cases had metastases recorded, the most common sites being the liver (32.6%), brain (11.1%) and bone (10.4%). Approximately 3% of confirmed cases had a record of prior asbestos exposure.

**Table 2:** Characteristics of confirmed cases of SCLC following the case validation exercise

Characteristic	No. of patients *N* = 381 n (%)
Recorded symptoms	322 (84.5)
Haemoptysis	38 (11.8)
Chest infection	58 (18.0)
Cough	51 (15.8)
Shortness of breath	37 (11.5)
Chest, abdominal or back pain	49 (15.2)
COPD/respiratory complications	41 (12.7)
Abnormal weight loss	13 (4.0)
Indirect finding	35 (10.9)
First diagnostic procedure recorded	278 (73.0)
Chest X-ray	182 (65.5)
CAT scan	49 (17.6)
Abdominal ultrasound	5 (1.8)
Bronchoscopy	33 (11.9)
Biopsy	5 (1.8)
Other	4 (1.4)
Stage	
Known	242 (63.5)
Limited	55 (23.7)*
Extensive	187 (76.3)*
Unknown	139 (36.5)
Chemotherapy/radiotherapy	
Chemotherapy	212 (55.6)
Radiotherapy	120 (31.5)
Metastasis	144 (37.8)
Asbestos exposure	11 (2.9)

*Percentage of the 242 cases with recorded stage

Computed tomography scan; CAT, *COPD* chronic obstructive pulmonary disease, *SCLC* small cell lung cancer

### Survival rates

There were a total of 2840 SCLC cases available for the survival analysis. Of these, a total of 1998 (70.4%) died within the first year following diagnosis; the 1-year crude mortality rate was 9.9 (95% CI: 9.5–10.4) per 100 person-months. Mortality was higher in men than women and increased with age (Table 3). Kaplan–Meier survival curves of cumulative incidence of death are shown in Additional file 1: Figures S1–S3). Median survival among the whole study cohort was 7.37 months.

**Table 3:** Cumulative 1-year all-cause mortality and all-cause mortality rates per 100 person-months in patients with newly-diagnosed SCLC, stratified by age and sex

		Sex		Age group (years)			
	Total	Male	Female	< 60	60–69	70–79	≥80
Individuals at risk, n	2840	1468	1372	536	948	1011	345
Deaths, %	1998	1081	917	333	632	753	280
Cumulative mortality, % (95% CI)	82.6 (81.1–84.1)	84.8 (82.8–86.73)	80.1 (77.8–82.4)	76.6 (72.6–80.5)	80.0 (77.1–82.7)	85.4 (83.0–87.6)	89.6 (85.9–92.7)
Person-months of observation	20,073	9787	10,287	4399	7110	6603	1961
IR per 100 person-months (95% CI)	9.95 (9.53–10.39)	11.05 (10.41–11.72)	8.91 (8.36–9.51)	7.57(6.80–8.43)	8.89 (8.22–9.61)	11.40 (10.62–12.25)	14.28 (12.70–16.05)

*CI* confidence interval, *IR* incidence rate, *SCLC* small cell lung cancer

## Discussion

In this study, we have investigated the feasibility of using a database of routinely collected primary care medical records to identify and characterise incident cases of SCLC in the UK. The high PPV found from our validation exercise suggests that our multistep strategy, involving use of text data mining in the free text comments of individuals’ EHRs, is a valid and effective method of identifying incident SCLC cases. The level of recording of clinical information among SCLC cases appears to be high, with the majority of cases having symptoms recorded prior to their diagnosis. Our study clearly demonstrates that use of the information entered by PCPs in the free text sections is imperative to identify and characterise patients with SCLC using THIN and other similar databases of EHRs. Although a time-consuming process, the importance of analysing free text information in THIN has been shown in previous studies. [13–15]

Among SCLC cases with a recorded disease stage, the majority (82.2%) had extensive disease. While most patients were expected to have extensive stage disease, this proportion is higher than reports from other studies in the UK and elsewhere. In the National Lung Cancer Audit of 18,513 patients with histologically proven SCLC (2004–2011), 67.2% of patients with recorded stage had extensive disease, albeit 20% of patients had no recorded stage. [6] In our study, just under a third of incident SCLC cases had no disease stage recorded, indicating that access to further data sources, such as cancer registries, would be required for full characterisation of this patient population. Using our case identification algorithm, incident cases of SCLC accounted for 11.7% of all patients initially detected with a Read code for either lung cancer (unspecified or specific of SCLC) or SCC. The true proportion of all lung cancer patients in the THIN source population that were cases of SCLC could be lower than this – because a small number of these initially detected patients may have been cases of small cell renal carcinoma – or higher if some cases of SCLC were missed owing to lack of data on morphology. This proportion, however, is in line with analyses of UK registry data from the UK National Lung Cancer Audit (10.3%) [6] and the Thames Cancer Registry (10–12%) [9] over earlier time periods, and similar to analyses of national lung cancer registrations in Denmark (15%) [4] and the US (13%). [5] While the similar age at SCLC diagnosis in our study cohort with that seen in the National Lung Cancer Audit (68.5 years vs. 70 years) supports the validity of our SCLC case identification method, the proportion of patients with a record of having received chemotherapy was lower (56% vs. 69%), again indicating that access to additional data sources; for example, the UK National Health Service’s Systematic Anti-Cancer Therapy chemotherapy dataset, may be required for a more complete characterisation of patients disease profile and treatments prescribed.

Data on the incidence of SCLC are scarce. Cancer registry data from the National Cancer Institute Surveillance, Epidemiology, and End Results (NCI SEER) program in the US reported the age-adjusted incidence of SCLC between 2010 and 2014 to be 0.65 per 10,000 person-years, slightly lower than our estimate of 1.01 per 10,000 person-years seen in a more recent time period. [16] Previous estimates of the incidence of SCLC in England and other European countries have also been lower, albeit from analysis of data collected more than a decade ago. [17] In their analysis of cancer registration data from the South East of England Thames Cancer Registry), Riaz et al, [9] reported age-standardized incidence rates of SCLC in 2007 of 0.6 per 10,000 in males and 0.4 per 10,000 in females. These slightly lower rates could reflect differences between the data collection methods in THIN and UK cancer registries. In an earlier period also using cancer registrations from the EUROCARE database (1993–1997), the age-standardized incidence of SCLC in the England was estimated as 0.56 per 10,000 person-years, which was one of the highest in Europe along with Denmark (0.79 per 100,000 person-years), Iceland (0.77 per 10,000 person-years) and Scotland (1.1 per 100,000 person-years). [17]

As expected, survival was poor in our cohort of incident SCLC cases and was worse in men and the elderly; median survival was 7.4 months in line with estimates from the National Lung Cancer Audit and NCI SEER program in the US. [18] Patient comorbidity can affect survival – analysis of data from the SCLC cancer registrations in the Eindhoven region of the Netherlands [10] showed that among those with limited stage disease, cardiac disease negatively affected prognosis while digestive disease was favourable for survival, and, among those with extensive-stage disease, prognosis was negatively affected by both cardiac and cerebrovascular diseases. We were able to describe prevalence of a wide range of comorbidities and comedications in our study, as well as lifestyle factors, using the routinely collected patient data recorded by PCPs. Cigarette smoking is a strong risk factor for SCLC [19] and, as expected, we showed the prevalence of smoking to be very high among our cohort of SCLC patients, with 94% being current or former smokers. The ability of THIN to describe clinical and lifestyle characteristics is a feature that complements the valuable data available in UK cancer registries. These complementary aspects of THIN would be even more beneficial if THIN was linked to UK cancer registries. However, at present, unlike a subset of practices in the Clinical Practice Research Datalink primary care database (a similar database to THIN but where free text comments are unavailable), there is no such linkage.

## Conclusion

In conclusion, we have developed a bespoke and valid method of identifying cases of SCLC in the UK using routinely-collected primary care EHRs, which could potentially be used to evaluate trends in incidence and survival. It could also potentially be modified to identify and analyse trends in other cancer types. While our method enables a good characterisation of patients’ comorbidity profile, a more complete characterisation of these patients’ disease, including stage and treatments received, would require access to additional data sources. An awareness of any future changes in coding methods, such as the introduction for specific Read codes for SCLC and linguistics used in free text entries, is also important to identify parts of our algorithm that may need to be adapted for future use.

## Additional file

Additional file 1: **Figure S1.** Kaplan–Meier cumulative survival estimates for the cohort of patients with newly diagnosed SCLC. **Figure S2.** Kaplan–Meier cumulative survival estimates for the cohort of patients with newly diagnosed SCLC, stratified by sex. **Figure S3.** Kaplan–Meier cumulative survival estimates for the cohort of patients with newly diagnosed SCLC, stratified by age. **Table S1.** Read codes suggestive of lung cancer of small cell cancer. **Table S2.** Read codes and additional health data codes used to request free text comments for manual review **Table S3.** Case classification after manual review of patient profiles with free text comments for the sample of 400 patients. **Table S4**. Positive predictive value of SSc in THIN by age, sex and smoking status following the manual review process including the free text. (DOCX 1789 kb)

## Abbreviations

CI: Confidence interval
IQR: Interquartile range
NCI SEER: National Cancer Institute Surveillance, Epidemiology, and End Results program
NSCLC: Non-small-cell lung cancer
PCP: Primary care practitioner
SCLC: Small-cell lung cancer
SD: Standard deviation
THIN: The Health Improvement Network
UK: United Kingdom
