# Supplementary material for: Feasibility and validity of The Health Improvement Network database of primary care electronic health records to identify and characterise patients with small cell lung cancer in the United Kingdom
Source: BMC Cancer. 2019 Jan 21;19:91. doi: 10.1186/s12885-019-5305-1 (PMC6341576; doi:10.1186/s12885-019-5305-1)
Supplement: Supplementary file 1 — Figure S1. Kaplan–Meier cumulative survival estimates for the cohort of patients with newly diagnosed SCLC. Figure S2. Kaplan–Meier cumulative survival estimates for the cohort of patients with newly diagnosed SCLC, stratified by sex. Figure S3. Kaplan–Meier cumulative survival estimates for the cohort of patients with newly diagnosed SCLC, stratified by age. Table S1. Read codes suggestive of lung cancer of small cell cancer. Table S2. Read codes and additional health data codes used to request free text comments for manual review Table S3. Case classification after manual review of patient profiles with free text comments for the sample of 400 patients. Table S4. Positive predictive value of SSc in THIN by age, sex and smoking status following the manual review process including the free text. (DOCX 1789 kb) [file 12885_2019_5305_MOESM1_ESM.docx]

**
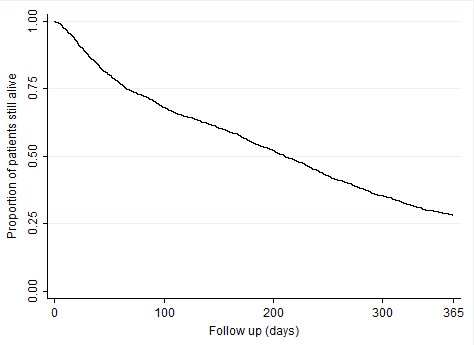
**

**Additional file 1: Figure S1.** Kaplan–Meier cumulative survival estimates for the cohort of patients with newly diagnosed SCLC.

**
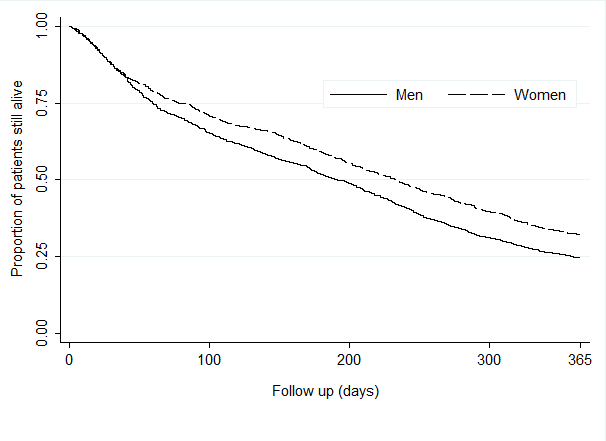
**

**Additional file 1: Figure S2.** Kaplan–Meier cumulative survival estimates for the cohort of patients with newly diagnosed SCLC, stratified by sex.

**
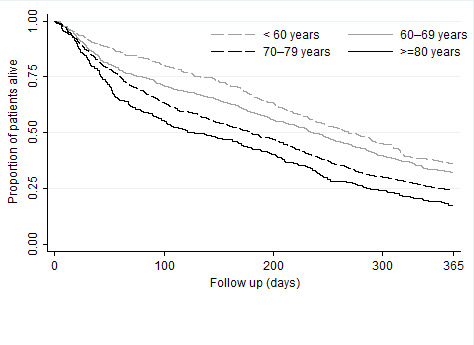
**

**Additional file 1: Figure S3.** Kaplan–Meier cumulative survival estimates for the cohort of patients with newly diagnosed SCLC, stratified by age.

**Additional file 1: Table S1.** Read codes suggestive of lung cancer of small cell cancer.

| **Read code** | **Description** |
| --- | --- |
| B221.00 | Malignant neoplasm of main bronchus |
| B221000 | Malignant neoplasm of carina of bronchus |
| B221100 | Malignant neoplasm of hilus of lung |
| B221z00 | Malignant neoplasm of main bronchus NOS |
| B222.00 | Malignant neoplasm of upper lobe, bronchus or lung |
| B222000 | Malignant neoplasm of upper lobe bronchus |
| B222100 | Malignant neoplasm of upper lobe of lung |
| B222z00 | Malignant neoplasm of upper lobe, bronchus or lung NOS |
| B223.00 | Malignant neoplasm of middle lobe, bronchus or lung |
| B223000 | Malignant neoplasm of middle lobe bronchus |
| B223100 | Malignant neoplasm of middle lobe of lung |
| B223z00 | Malignant neoplasm of middle lobe, bronchus or lung NOS |
| B224.00 | Malignant neoplasm of lower lobe, bronchus or lung |
| B224000 | Malignant neoplasm of lower lobe bronchus |
| B224100 | Malignant neoplasm of lower lobe of lung |
| B224z00 | Malignant neoplasm of lower lobe, bronchus or lung NOS |
| B225.00 | Malignant neoplasm of overlapping lesion of bronchus & lung |
| B22y.00 | Malignant neoplasm of other sites of bronchus or lung |
| B22z.00 | Malignant neoplasm of bronchus or lung NOS |
| B22z.11 | Lung cancer |
| B812.00 | Carcinoma in situ of bronchus and lung |
| B812000 | Carcinoma in situ of carina of bronchus |
| B812100 | Carcinoma in situ of main bronchus |
| B812200 | Carcinoma in situ of upper lobe bronchus and lung |
| B812300 | Carcinoma in situ of middle lobe bronchus and lung |
| B812400 | Carcinoma in situ of lower lobe bronchus and lung |
| B812z00 | Carcinoma in situ of bronchus or lung NOS |
| B23..00 | Malignant neoplasm of pleura |
| B230.00 | Malignant neoplasm of parietal pleura |
| B231.00 | Malignant neoplasm of visceral pleura |
| B23y.00 | Malignant neoplasm of other specified pleura |
| B23z.00 | Malignant neoplasm of pleura NOS |
| B908000 | Neoplasm of uncertain behaviour of pleura |
| Byu2100 | [X]Malignant neoplasm/overlap lesion/heart,mediastinm+pleura |
| B22..00 | Malignant neoplasm of trachea, bronchus and lung |
| B220.00 | Malignant neoplasm of trachea |
| B220000 | Malignant neoplasm of cartilage of trachea |
| B220100 | Malignant neoplasm of mucosa of trachea |
| B220z00 | Malignant neoplasm of trachea NOS |
| B907.00 | Neoplasm of uncertain behaviour trachea, bronchus and lung |
| B907000 | Neoplasm of uncertain behaviour of trachea |
| B907z00 | Neop of uncertain behaviour of trachea, bronchus or lung NOS |
| B907100 | Neoplasm of uncertain behaviour of bronchus |
| B907200 | Neoplasm of uncertain behaviour of lung |
| BB08.00 | [M]Malignant tumour, small cell type |
| BB1J.00 | [M]Small cell carcinoma NOS |
| BB1L.00 | [M]Small cell carcinoma, fusiform cell type |
| BB1M.00 | [M]Small cell carcinoma, intermediate cell |
| BB1N.00 | [M]Small cell-large cell carcinoma |
| BB1P.00 | [M]Non-small cell carcinoma |
| BB1K.00 | [M]Oat cell carcinoma |

**Additional file 1: Table S2.** Read codes and additional health data codes used to request free text comments for manual review

| **Read code** | **Description** |
| --- | --- |
| B221.00 | Malignant neoplasm of main bronchus |
| B221000 | Malignant neoplasm of carina of bronchus |
| B221100 | Malignant neoplasm of hilus of lung |
| B221z00 | Malignant neoplasm of main bronchus NOS |
| B222.00 | Malignant neoplasm of upper lobe, bronchus or lung |
| B222000 | Malignant neoplasm of upper lobe bronchus |
| B222100 | Malignant neoplasm of upper lobe of lung |
| B222z00 | Malignant neoplasm of upper lobe, bronchus or lung NOS |
| B223.00 | Malignant neoplasm of middle lobe, bronchus or lung |
| B223000 | Malignant neoplasm of middle lobe bronchus |
| B223100 | Malignant neoplasm of middle lobe of lung |
| B223z00 | Malignant neoplasm of middle lobe, bronchus or lung NOS |
| B224.00 | Malignant neoplasm of lower lobe, bronchus or lung |
| B224000 | Malignant neoplasm of lower lobe bronchus |
| B224100 | Malignant neoplasm of lower lobe of lung |
| B224z00 | Malignant neoplasm of lower lobe, bronchus or lung NOS |
| B225.00 | Malignant neoplasm of overlapping lesion of bronchus & lung |
| B22y.00 | Malignant neoplasm of other sites of bronchus or lung |
| B22z.00 | Malignant neoplasm of bronchus or lung NOS |
| B22z.11 | Lung cancer |
| B812.00 | Carcinoma in situ of bronchus and lung |
| B812000 | Carcinoma in situ of carina of bronchus |
| B812100 | Carcinoma in situ of main bronchus |
| B812200 | Carcinoma in situ of upper lobe bronchus and lung |
| B812300 | Carcinoma in situ of middle lobe bronchus and lung |
| B812400 | Carcinoma in situ of lower lobe bronchus and lung |
| B812z00 | Carcinoma in situ of bronchus or lung NOS |
| B23..00 | Malignant neoplasm of pleura |
| B230.00 | Malignant neoplasm of parietal pleura |
| B231.00 | Malignant neoplasm of visceral pleura |
| B23y.00 | Malignant neoplasm of other specified pleura |
| B23z.00 | Malignant neoplasm of pleura NOS |
| B908000 | Neoplasm of uncertain behaviour of pleura |
| Byu2100 | [X]Malignant neoplasm/overlap lesion/heart,mediastinm+pleura |
| B22..00 | Malignant neoplasm of trachea, bronchus and lung |
| B220.00 | Malignant neoplasm of trachea |
| B220000 | Malignant neoplasm of cartilage of trachea |
| B220100 | Malignant neoplasm of mucosa of trachea |
| B220z00 | Malignant neoplasm of trachea NOS |
| B907.00 | Neoplasm of uncertain behaviour trachea, bronchus and lung |
| B907000 | Neoplasm of uncertain behaviour of trachea |
| B907z00 | Neop of uncertain behaviour of trachea, bronchus or lung NOS |
| B907100 | Neoplasm of uncertain behaviour of bronchus |
| B907200 | Neoplasm of uncertain behaviour of lung |
| BB08.00 | [M]Malignant tumour, small cell type |
| BB1J.00 | [M]Small cell carcinoma NOS |
| BB1L.00 | [M]Small cell carcinoma, fusiform cell type |
| BB1M.00 | [M]Small cell carcinoma, intermediate cell |
| BB1N.00 | [M]Small cell-large cell carcinoma |
| BB1P.00 | [M]Non-small cell carcinoma |
| 6A...00 | Patient reviewed |
| 6A1..00 | Patient reviewed at hospital |
| 744Bz11 | Bronchoscopy NEC |
| 7M37100 | Radiotherapy NEC |
| 8BAD.00 | Chemotherapy |
| 8BAV.00 | Cancer care review |
| 8BC1.00 | Treatment plan given |
| 8CM1.00 | On gold standards palliative care framework |
| 8H3Z.00 | Other hospital admission NOS |
| 8HE..00 | Discharged from hospital |
| 93A..00 | Discharge summary |
| 9N09.00 | Seen in oncology clinic |
| 9N0M.00 | Seen in radiology department |
| 9N19.00 | Seen in hospital casualty |
| 9N1C.11 | Home visit |
| 9N1y800 | Seen in oncology clinic |
| 9N2g.00 | Seen by respiratory physician |
| 9N31.00 | Telephone encounter |
| 9ND..11 | Incoming mail |
| 9ND4.00 | X-ray report received |
| 9NDF.00 | Fax received |
| 9NDZ.00 | Incoming mail NOS |
| 9Z...00 | Administration NOS |
| Z172.00 | Palliative care |
| ZV57C00 | [V]Palliative care |
| 9N36.00 | Letter from specialist |
| 9ND6.00 | Communication from: |
| **AHD** | **Descriptor** |
| 1001400091 | Chest X-ray |
| 1001400166 | Bronchoscopy |
| 1001400172 | CAT scan |
| 1001400164 | MRI scan |

**Additional file 1: Table S3.** Case classification after manual review of patient profiles with free text comments for the sample of 400 patients.

| **Case classification** | **Group 1^*^**  **N=300**  **n (%)** | **Group 2^†^**  **N=100**  **n (%)** |
| --- | --- | --- |
| Confirmed SCLC  Non-case  Non-small cell lung cancer  Lung cancer as secondary tumour  Other localization  Diagnosed before start date  Non-confirmed | **296 (98.7)**  4 (1.3)  4 (100.0)  –  –  –  – | **85 (85.0)**  15 (15.0)  10  2  1  1  1 |

**^*^**Sample of 300 patients identified with a Read code suggestive of lung cancer in the first step of the SCLC case identification process, and classed as an expected case based on subsequent case identification steps. **^†^** Sample of 100 patients identified with a Read code suggestive of small cell cancer in the first step of the SCLC case identification process, and classed as an expected case based on subsequent case identification steps.

**Additional file 1: Table S4**. PPV of SCLC in THIN based on findings from the manual review of patient records.

| **Characteristic** | **Positive predictive value of SCLC in THIN using our algorithm, %** |
| --- | --- |
| **Sex** |  |
| Men | 94.5 (208/220) |
| Women | 96.1 (173/180) |
| **Age group (years)** |  |
| 40–49 | 93.8 (15/16) |
| 50–59 | 90.0 (45/50) |
| 60–69 | 95.5 (128/134) |
| 70–79 | 95.8 (138/144) |
| ≥80 | 98.2 (55/56) |
| **Smoking** |  |
| Current smoking | 92.6% (25/27) |
| Non-smoking | 95.8 (183/191) |
| Former smoking | 95.5 (168/176) |
| Unknown smoking status | 83.3 (5/6) |

PPV, positive predictive value; SCLC, small-cell lung cancer; THIN, The Health Improvement Network.
